# Notes from London

**Published:** 1897-07

**Authors:** J. L. Campbell

**Affiliations:** Demonstrator of Anatomy, Atlanta Medical College; London, Eng.


### CORRESPONDENCE.

### NOTES FROM LONDON.

LONDON, ENG., May 27, 1897.

Surgical work is at the height of the season now I should say, judging from the amount that is being done at the various hospitals, and for the most part is it all major work. I have seen some of the best surgeons here operate, and they are good, practical men.

I have seen quite a number of operations for radical cure of hernia. I like the operation that Mr. Lockwood does at St. Bartholomew's. It seems to be something like Bassini's operation. He cuts down on the sack in the usual way, dissects it out, then twists it several times, after opening it to see that all the intestines or omentum have gone back, transfixes the twisted sack close up, ties it, then brings one of the ligatures quite around again, and ties one more time; cuts off the sack, lets the stump fall back into the cavity, then threads the two ends of the ligature in curved needles and fixes the stump to the abdominal wall by passing the needles through the transversalis, internal and external oblique muscles at the internal ring, and then ties it firmly. He then raises the cord, holds it to the upper angle of his wound, sutures the conjoined tendon of the transversalis and internal oblique to Poupart's ligament, making a new posterior wall for the inguinal canal and sutures up the aponeurosis of the external oblique for the new anterior wall, closes the wound, puts a drainage-tube in the lower angle, and dresses it in the usual way.

Mr. Marsh does the operation in a little different way, passing the two ends of the ligature of the sack through the muscles, one above and the other below the incision. He uses kangaroo tendon instead of silk.

At the London Hospital, where I am studying, I have seen some very good work both in medicine and surgery. I have seen

Mr. Treves operate several times. He does a very pretty operation for appendicitis. He believes that you should always wait until the acute attack has subsided. If there is an abscess formed and points open it and the patient will not have a second attack. In relapsing cases of appendicitis, try to operate between the attacks. I saw him do two on Friday, and heard him say that out of one hundred and seventy-two cases he had operated on, that he had had only one death. If Mr. Treves can get such results on the class of patients that he has at the London Hospital, there is no reason why surgeons in Georgia should not do as well or better on the comparatively good class of patients that we have. Mr. Treves is a fast operator, and keeps his patients under the anesthetic just as short a time as he can. He makes a rapid incision to the peritoneal cavity, then introduces his finger, lifts up the first portion of the colon that he can, then follows it until he reaches the appendix. In this way he finds it very quickly, breaks up any adhesions that may exist, ligates the mesentery and the appendix, removes it, swabs the ends with a very strong solution of carbolic acid, or touches the end with pure carbolic acid, then puts two sutures through the stump, ties them moderately tight, then closes the peritoneum with silk, and the skin with silkworm gut.

I saw Mr. F. S. Eve do a Kraske operation, or a modification of the original Kraske operation, on a case of carcinoma of the rectum. The patient was a man, thirty-three years of age, in moderately good physical condition. Had only noticed blood in his stools for two or two and a half months. The cancer was high up and all the structures considerably infiltrated. He did not have any obstruction, so there was no colotomy performed. He cut down on the sacrum, going from just a little behind the margin of the anus to the level of the three posterior sacral foramina, then making an incision at right angles to the first at this point, dissected the muscles from the bone, then sawed through the bone in the median line, and across at the third sacral vertebræ, retracted the bone and exposed the rectum. It was very much adherent to all the tissues, and took some time to get up to the sigmoid flexure. He brought the gut down, and making an incision about three inches above the anus, removed about four inches of the rectum above this, brought the proximal end down and sutured it to the remaining end of the rectum. Before doing this, however, he closed the peritoneal cavity with fine silk. He then replaced the bones, suturing them with heavy silk through the periosteum, and closed the wound with silkworm gut, putting in a large drainage-tube. The patient did well for a few days, when he had considerable rise of temperature, but nothing could be found in the wound, which was opened and packed with iodoform gauze. It was found that he had pneumonia, which was treated with oxygen and general stimulants, but he died after eighteen or twenty days. At the post-mortem it was found that the wound in the rectum was united perfectly, union by first intention; one lung was in the stage of gray hepatization, the other red hepatization, while he had a rapid recurrence of the carcinoma in his liver and mesenteric glands, many of which were involved. Mr. Eve had done two cases before in which he had to do colotony first, and in these cases he found that by not removing the sacrum, as is advised in the books, the patients were able to sit down with comfort after the operation.

J. L. CAMPBELL, M.D.,
Demonstrator of Anatomy, Atlanta Medical College.

